# Protective Effects of Inflammation of Curcumae Longae Rhizoma 30% EtOH Extract on Acute Reflux Esophagitis Rats

**DOI:** 10.1155/2021/8854945

**Published:** 2021-01-16

**Authors:** Jin A. Lee, Mi-Rae Shin, Min Ju Kim, Ji Hye Lee, Hae-Jin Park, Seong-Soo Roh

**Affiliations:** ^1^Department of Herbology, College of Korean Medicine, Daegu Haany University, 136, Shinchendong–ro, Suseong-gu, Daegu 42158, Republic of Korea; ^2^Herbal Medicine Resources Research Center, Korea Institute of Oriental Medicine, 111, Geonjae-ro, Naju-si, Jeollanam-do 58245, Republic of Korea; ^3^DHU Bio Convergence Testing Center, 1, Hanuidae-ro, Gyeongsan-si, Gyeongsangbuk-do 38610, Republic of Korea

## Abstract

Gastroesophageal reflux disease (GERD) is induced by the reflux of stomach contents or gastric acid, pepsin into the esophagus for prolonged periods of time due to defection of the lower esophageal sphincter. Reflux esophagitis is a disease found in less than 50% of GERD patients. This study is aimed at evaluating the protective effect of Curcumae longae Rhizoma 30% EtOH extract (CLR) in acute reflux esophagitis (ARE) rats. CLR measured antioxidant activity through *in vitro* experiments. Based on the results, we performed experiments *in vivo*. Before 90 min ARE induction, CLR was administered orally by concentration. ARE was derived by linking the metastatic junction between pylorus and forestomach and corpus in Sprague-Dawley rats. And rats were sacrificed 5 h after surgery. We analyzed the expression of antioxidant and inflammatory-related markers by western blot and observed the production of alanine aminotransferase (ALT), aspartate aminotransferase (AST), reactive oxygen species (ROS), peroxynitrite (ONOO^−^), and thiobarbituric acid reactive substance (TBARS). The administration of CLR reduced esophagus tissue damage in rats with acute reflux esophagitis and decreased the elevated ALT, AST, ROS, ONOO^−^, and TBARS. In addition, CLR effectively increased antioxidant-related factors and reduced inflammatory protein. Overall, these results suggest that CLR would be used as a therapeutic material in protection and treatment for ARE. Overall, CLR treatment informed that markedly ameliorated inactivation of NF-*κ*B led to the inhibition of the expressions of proinflammatory proteins. These results suggest that CLR would be used as a therapeutic material in protection and treatment for ARE.

## 1. Introduction

Gastroesophageal reflux disease (GERD) is a chronic disease involving epithelial metaplasia and mucosal damage [1]. GERD is induced by the reflux of stomach contents or gastric acid, pepsin into the esophagus for prolonged periods of time due to a defection of the lower esophageal sphincter [2, 3]. In 2005, the percentage of GERD prevalence in Eastern Asia was 2.5–4.8%. Thereafter, from 2005 to 2010, it increased to 6.3–18.3% in Southeast and Western Asia and to 5.2–8.5% in Eastern Asia [4]. Reflux esophagitis (RE), a mild to moderate GERD, is a disease found in less than 50% of GERD patients [5]. Recent studies have shown that RE is mediated by oxygen-induced free radicals [6]. Oxidative stress could lead to leukocyte activation, ROS production, and increased tissue damage [7]. Overproduction of ROS such as superoxide hydrogen peroxide (H_2_O_2_), hydroxyl radicals (·OH), and anions (O_2_−) is overexpressed in inflammatory gastroesophageal tissues, and can contribute to the immediate development of inflammatory processes [8].

Current management of RE includes the use of antisecretory treatments aimed primarily at reducing gastric acidity, such as the proton pump inhibitors (PPIs), antacids, or histamine-receptor antagonists. In particular, acid suppression achieved with PPIs is the mainstay of therapy for reflux disease, but despite this, symptoms and damage persist and recur in many patients. Also, despite their well-known health effects, these therapies could determine the development of severe complications, relapse, and various adverse effects because of the long-term use [9, 10]. As an alternative to these problems, in recent years, the development of therapeutic agents using natural materials has been actively conducting [11–13].

Curcumae longae Rhizoma, a rootstock plant belonging to Zingiberaceae, is extensively cultivated in the tropical areas of Asia, and has been widely used for medical treatments of various diseases in Asian countries mostly. Curcumae longae Rhizoma is known to protect the gastric mucosa by reducing acid release and increasing mucus production, and has been used as a treatment for peptic ulcer, especially in Korean medicine. The Ayurvedic Indian medicine claims the use of Curcumae longae Rhizoma against biliary disorders, stomach tumor. Besides that, it has been used as an anti-inflammatory to treat diseases such as colic, chest pain, and menstrual disorders [14, 15]. Curcumin, the active ingredient of Curcumae longae Rhizoma, is a lipophilic polyphenolic substance. Polyphenolic substances such as curcumin have been found to exhibit antioxidant, anti-inflammatory, and antibacterial effects [15, 16]. Also, curcumin treatment has also been shown to be effective in improving immune kidney disease, diabetes, and cardiovascular disease, and has been shown to act as a protective agent in gastroesophageal disease by protecting tissue damage, especially by improving inflammation and apoptosis [17]. Inflammation, which is the compensatory response about tissue injury, is induced in the increase of leukocytes and inflammatory factors such as cytokines and chemokines [18]. In the damaged esophageal mucosa in RE, an inflammatory reaction occurs by activation of NF-*κ*B, a major regulator of inflammatory proteins, and can lead to chronic disease if excessively progressed [2, 19].

The purpose of our study was to demonstrate the relationship between inflammatory response and esophageal mucosal damage through inhibition of NF-*κ*B activation in reflux esophagitis rats administered with Curcumae longae Rhizoma.

## 2. Materials and Methods

### 2.1. Materials

L-(+)-ascorbic acid and diethylene glycol were purchased from Alfa Aesar (Lancashire, UK). Folin-Ciocalteu's phenol reagent 2,2-diphenyl-1-picrylhydrazyl, potassium persulfate, 2,2′-azino-bis(3-ethylbenzothiazoline-6-sulfonic acid) diammonium salt, 2-thiobarbituric acid, gallic acid, naringin, phenyl methane sulfonyl fluoride (PMSF), and 1,1,3,3-tetramethoxypropane were purchased from Sigma-Aldrich (St. Louis, MO, USA). Sodium carbonate was purchased from Daejung Chemicals & Metals Co., Ltd. (Siheung, Korea). Sodium hydroxide was purchased from OCI Company Ltd. (Seoul, Korea). Phosphoric acid was purchased from Duksan Company (Ansan, Korea). 2′,7′-Dichloro fluorescein diacetate (DCF-DA) was purchased from Molecular Probes (Eugene, OR, USA). The protease inhibitor mixture solution and ethylenediaminetetraacetic acid (EDTA) were purchased from Wako Pure Chemical Industries, Ltd. (Osaka, Japan). The pierce BCA protein assay kit was purchased from Thermo Fisher Scientific (Waltham, MA, USA). ECL Western Blotting Detection Reagents and pure nitrocellulose membranes were purchased from GE Healthcare (Chicago, IL, USA). Rabbit polyclonal antibodies against nuclear factor erythroid-derived 2-related factor 2 (Nrf2), NADPH oxidase 4 (NOX4), p22^phox^, superoxide dismutase (SOD), catalase, glutathione peroxidase-1/2 (GPx-1/2), c-Jun N-terminal kinase (JNK), and inhibitor of nuclear factor kappa B alpha (I*κ*B*α*); goat polyclonal antibodies against tumor necrosis factor-alpha (TNF-*α*) and interleukin-6 (IL-6); and mouse polyclonal antibodies against phosphor-JNK (p-JNK), nuclear factor-kappa B p65 (NF-*κ*Bp65), phosphoinhibitor of nuclear factor kappa B alpha (p-I*κ*B*α*), inducible nitric oxide synthase (iNOS), cycloxygenase-2 (COX-2), claudin-3, claudin-4, histone, and beta-actin (*β*-actin) were purchased from Santa Cruz Biotechnology, Inc. (Dallas, TX, USA). Also, rabbit polyclonal antibodies against phospho-p38 (p-p38) and mouse polyclonal antibodies against c-Jun were purchased from Cell Signaling Technology (Danvers, MA, USA). And goat anti-rabbit, rabbit anti-goat, and goat anti-mouse immunoglobulin G (IgG) horseradish peroxidase- (HRP-) conjugated secondary antibodies were purchased from GeneTex, Inc. (Irvine, LA, USA).

### 2.2. Preparation of the Plant Material

Curcumae longae Rhizoma, native to India, was purchased from Daemyung Pharm. Co. Ltd. (Seoul, Korea). A voucher herbarium specimen was verified at the College of Korean Medicine in Daegu Haany University. 100 g of herb dried Curcumae longae Rhizoma was extracted with 10 times EtOH: distilled water (3 : 7) for 24 hours at room temperature. The mixture was concentrated in evaporated *in vacuo* and dried completely using a freeze dryer to obtain a powder (CLR; the yield rate of 7.1%). Powder is stored at -80°C until animal experimentation.

### 2.3. CLR Analysis by HPLC Chromatogram

**Figure 1:**
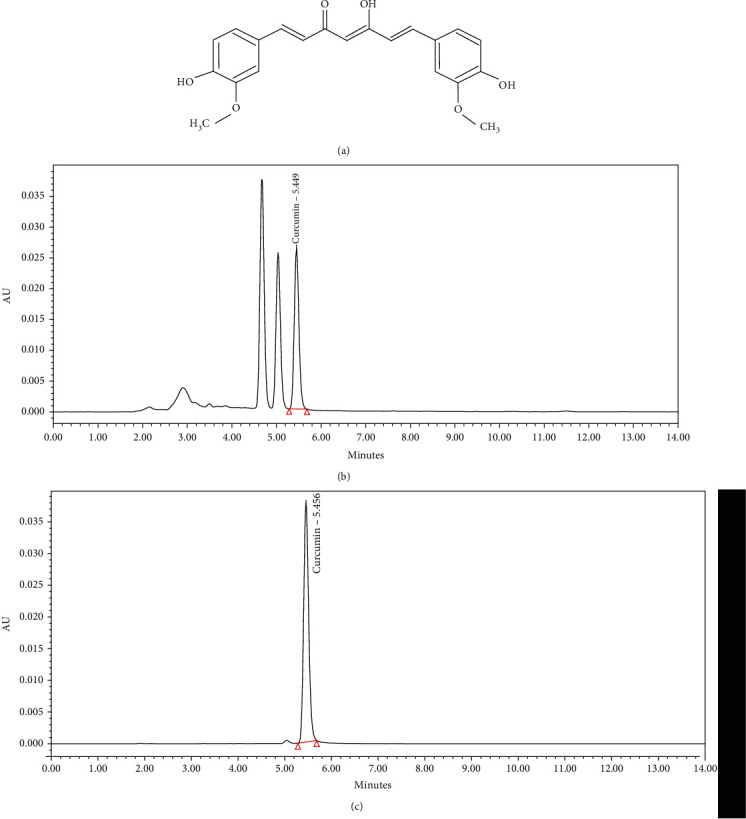
HPLC profile of Curcumae longae Rhizoma 30% EtOH extract (CLR). Curcumin (C_21_H_20_O_6_: 368.38 g/mol) (a), HPLC profile of curcumin;(b), and HPLC profile of Curcumae longae Rhizoma 30% EtOH extract (c).

The extract of CLR (1 mg) was dissolved in 1 mL of 70% methanol. 10 *μ*L of sample was injected into high-performance liquid chromatography (HPLC) using YMC-Pack Pro C18 RS (4.6 × 150 mm, 5 *μ*m). The mobile phase was composed of acetonitrile (A) and acetic acid (B), and its flow rate was 1.0 mL/min. The UV absorbance of 420 nm was monitored using a Waters e2695 (Water Corporation, MA, USA), and the column temperature was kept at a constant 25°C throughout the analysis. The peak areas were used to calculate the sample contents of the compounds. Representative HPLC results are illustrated in Figure 1. The amount was as follows: curcumin: 4.05 *μ*g/mL.

### 2.4. Total Polyphenol and Total Flavonoid Contents

Total polyphenol content was measured by the method of Folin and Denis [20]. 10 *μ*L of each sample was mixed with 790 *μ*L of distilled water and 50 *μ*L of Folin-Ciocalteu's phenol reagent, followed by reaction at room temperature (20°C) for 1 min, and then 150 *μ*L of 20% sodium carbonate was added. After reacting with 2 h at room temperature (20°C), absorbance was measured at 765 nm using a UV-VIS spectrophotometer, model infinite M200 Pro. Gallic acid was used to plot a standard calibration curve and calculate the total polyphenol content of the sample.

Total flavonoid content was measured by the method of Lister et al. [21]. 1 mL of diethylene glycol, 100 *μ*L of each sample, and 10 *μ*L of 1 N NaOH were mixed well and reacted at 37°C for 1 h, and then, the absorbance was measured at 420 nm using an UV-VIS spectrophotometer, model infinite M200 Pro. Naringin was used to plot a standard calibration curve and calculate the total flavonoid content of the sample.

### 2.5. DPPH Free Radical Scavenging Activity

The antioxidative effect of CLR was determined by the DPPH radical scavenging assay [22]. 100 *μ*L of CLR (blank; 100 *μ*L of distilled water) was added to equal volumes of an ethanolic solution of DPPH (60 *μ*M) in a 96-well microplate. L-ascorbic acid was used as a standard sample. The reaction mixtures were incubated at room temperature (20°C) for 30 min in the dark, and the optical density was determined using an UV-VIS spectrophotometer, model infinite M200 Pro (Tecan, Switzerland) at 540 nm. The antioxidant activity of each sample was expressed by IC_50_. The radical scavenging activity was calculated as % using the following equation:

DPPH radical scavenging activity (%) = {1 − (OD_blank_–OD_sample_/OD_blank_)} × 100.

### 2.6. ABTS Free Radical Scavenging Activity

The antioxidative effect of CLR was determined by the ABTS radical scavenging assay [23]. L-ascorbic acid was used as a standard sample. The ABTS solution was dissolved in water at 7.4 mM concentration. ABTS free radical cation (ABTS^+^) was produced by reacting ABTS stock solution with 2.45 mM and potassium sulfate and leaving the mixture in the dark for 16~18 h at room temperature (20°C). Calibrate the ABTS solution with ethanol to have an absorbance of 0.70 ± 0.02 at 415 nm. After adding 95 *μ*L of ABTS solution to 5 *μ*L of each of the sample, the mixture was left for 15 min at room temperature (20°C) in the dark. And the optical density was determined using an UV-VIS spectrophotometer, model infinite M200 Pro at 415 nm. The antioxidant activity of each sample was expressed by IC_50_. The radical scavenging activity was calculated as % using the following equation:

ABTS radical scavenging activity (%) = {1 − (OD_blank_–OD_sample_/OD_blank_)} × 100.

### 2.7. Acute Reflux Esophagitis Model

The animal experiments were performed according to the “Guidelines for Animal Experiment” approved by Ethics Committee of the Daegu Haany University (Approval No. DHU2019-128). 6-week-old male Sprague-Dawley rats (B.W. 180~200 g) were purchased from DaehanBiolink (Eumseong, Korea) and used for experiments after being adapted to environment for 1 week. Environmental conditions were set to 12 h light/dark cycle, controlled humidity (50 ± 5%), and temperature (22 ± 2°C). After 1 week adaptation, a total of 50 rats were randomly divided into 5 groups as follows:

- Nor: normal group
- Veh: water administered and acute reflux esophagitis-induced rats
- CL: CLR 50 mg/kg body weight administered and acute reflux esophagitis-induced rats
- CM: CLR 100 mg/kg body weight administered and acute reflux esophagitis-induced rats
- CH: CLR 200 mg/kg body weight administered and acute reflux esophagitis-induced rats

Rats were fasted 18 h prior to surgery and maintained with a raised mesh bottom cage to prevent copropagation, and water was supplied until surgery. 90 min before surgery, rats were orally administered with water or CLR. After that, rats were anesthetized by injection of 0.75 mg/kg doses of Zoletil (Virbac S.A. France) and expose the gastric gland and then a midline laparotomy was performed, which ligated both the pylorus and the transitional junction between the corpus and the forestomach with 2-0 silk thread by Omura et al. [24]. 5 h after surgery, rats were sacrificed to collect blood and esophageal tissue, and the esophageal tissues were immediately stored at −80°C.

### 2.8. Esophageal Ulcer Ratio

After sacrifice, the rat esophagus was cut from the gastroesophageal junction to the pharynx after sacrifice. The dissected esophagus was taken using an optical digital camera and then analyzed using the i-Solution Lite software program (Innerview Co., Korea).

The gross mucosal ulcer ratio (%) = [width of area with esophageal mucosal ulcer (mm^2^)/width of total area of the esophagus (mm^2^)] × 100.

### 2.9. Measurement of Gastric Acid pH

After sacrifice, the stomach of rat was washed with 1 mL of saline using a 1,000 *μ*L micropipette. The pH of the collected gastric juices was measured using a pH meter (EcoMet, iSTEK Co., Seoul, Korea).

### 2.10. Measurement of AST and ALT Levels in Serum

Hepatic functional parameters aspartate aminotransferase (AST) and alanine aminotransferase (ALT) assays were measured with a microplate fluorescence reader using a commercial kit (transaminase CII-test from Wako Pure Chemical Industries Ltd., Osaka, Japan).

### 2.11. Measurement of ROS and ONOO^−^ Levels in Serum

Reactive oxygen species (ROS), an oxidative stress biomarker, was measured according to the method of Ali et al. [25]. After mixing serum and 1 mM EDTA-50 mM sodium phosphate buffer (pH 7.4), 25 mM DCFH-DA was added, and after incubation for 30 min, the changes in fluorescence values were determined at emission of 535 nm and excitation of 485 nm using a UV-VIS spectrophotometer, model infinite M200 Pro (Tecan, Switzerland) every 5 min for 30 min.

Peroxynitrite (ONOO^−^), an oxidative stress biomarker, was measured according to the method of Kooy et al. [26]. After mixing serum and DHR123 buffer (rhodamine buffer, 5 mM DTPA, 10 mM DHR123) and after incubation for 5 min at 37°C, after that, the changes in fluorescence values were determined at emission of 535 nm and excitation of 485 nm using a UV-VIS spectrophotometer, model infinite M200 Pro (Tecan, Switzerland) every 5 min for 30 min.

### 2.12. Measurement of TBARS Levels in Serum and Tissue

The 2-thiobarbituric acid reactive substance (TBARS) levels were measured according to the method of Mihara and Uchiyama [27]. 1,1,3,3,-Tetramethoxypropane was used as a standard sample. After mixing samples and 1% phosphoric acid, 0.67% thiobarbituric acid was added, boiling for 45 min at 95°C. After that, mix butanol and centrifuge (3000 rpm, 10 min) to use it as a supernatant. Dispense the supernatant, and absorbance was measured at 540 nm using an UV-VIS spectrophotometer, model infinite M200 Pro (Tecan, Switzerland).

### 2.13. Preparation of Cytosol and Nuclear Factions

The extraction of protein was performed according to the method of Komatsu with modifications [28]. For cytosol fractions, esophageal tissues were homogenized with 250 mL ice-cold lysis buffer A containing 10 mM HEPES (pH 7.8), 10 mM KCl, 2 mM MgCl_2_, 1 mM DTT, 0.1 mM EDTA, 0.1 mM PMSF, and 1,250 *μ*L protease inhibitor mixture solution. The tissue homogenates were incubated (4°C for 20 min), and then, 10% NP-40 was mixed well. After centrifugation (13,400  ×  *g* at 4°C for 2 min) using Eppendorf 5415R (Hamburg, Germany), the supernatant (cytosol fractions) was separated with new Eppendorf tubes. The pellets were washed twice by the lysis buffer and discard the supernatant. After that, the pellets were suspended with 20 mL ice-cold lysis buffer C containing 300 mM NaCl, 50 mM HEPES (pH 7.8), 50 mM KCl, 1 mM DTT, 0.1 mM PMSF, 0.1 mM EDTA, 1% (*v*/*v*) glycerol, and 100 *μ*L protease inhibitor mixture solution which were suspended and incubated (4°C for 30 min). And in centrifugation (13,400  ×  *g*at 4°C for 10 min), the supernatant (nuclear fractions) was collected with new Eppendorf tubes. Both cytosol and nuclear fractions were stored at -80°C before the analysis.

### 2.14. Immunoblotting Analyses

For the estimation of Nrf2, c-Jun, NF-*κ*Bp65, and histone, 12 *μ*g of protein from each nuclear fraction was electrophoresed through 10% sodium dodecyl sulfate polyacrylamide gel (SDS-PAGE). Separated proteins were transferred to a nitrocellulose membrane, blocked with 5% (*w*/*v*) skim milk solution for 1 h, and then incubated with primary antibodies (Nrf2, c-Jun, NF-*κ*Bp65, and histone) overnight at 4°C. After the blots were washed, they were incubated with anti-rabbit or anti-mouse IgG HRP-conjugated secondary antibody for 1 h at room temperature. In addition, 8 *μ*g proteins of each cytosol fraction of NOX4, p22^phox^, SOD, catalase, GPx-1/2, p-p38, p-JNK, p-I*κ*B*α*, I*κ*B*α*, Inos, COX-2, TNF-*α*, IL-6, claudin-3, claudin-4, and *β*-actin were electrophoresed through 10-12% SDS-PAGE. Each antigen-antibody complex was visualized using ECL Western Blotting Detection Reagents and detected by chemiluminescence with Sensi-Q 2000 Chemidoc (Lugen Sci Co., Ltd., Gyeonggi-do, Korea). Band densities were measured using ATTO Densitograph Software (ATTO Corporation, Tokyo, Japan) and quantified as the ratio to histone or *β*-actin. The protein levels of the groups are expressed relative to those of the normal rat (represented as 1) [29].

### 2.15. Histological Examination

Histological examination through microscopy was performed to evaluate the separated esophagus tissues. The separated esophagus was fixed through a 10% neutral-buffered formalin and embedded in paraffin, and cut into 2 *μ*m sections and stained using hematoxylin and eosin (H&E) for microscopic evaluation. The stained slices were observed under an optical microscope and then analyzed using the i-Solution Lite software program (Innerview Co., Korea).

### 2.16. Statistical Analysis

*In vitro* values were expressed as the means ± SEM and *in vivo* values as the means ± SD. Statistical comparisons were analyzed by one-way ANOVA tests followed by the least significant difference (LSD) test using SPSS (version 25.0, IBM, Armonk, NY, USA). Values of *p* < 0.05 were considered significant.

## 3. Results

### 3.1. DPPH and ABTS Radical Scavenging Activity

The antioxidant effect of CLR was measured by DPPH radical scavenging assay, which is expressed in IC_50_ (*μ*g/mL). IC_50_ represents half the maximum concentration of compound tested to remove DPPH radicals. The IC_50_ value of L-ascorbic acid (standard sample) was 1.22 ± 0.04 *μ*g/mL, and the IC_50_ value of DPPH radical scavenging activity of CLR was 36.44 ± 0.76 *μ*g/mL. The antioxidant effect of CLR was measured by ABTS radical scavenging assay, which is expressed in IC_50_ (*μ*g/mL). IC_50_ represents half the maximum concentration of compound tested to remove ABTS radicals. The IC_50_ value of ABTS radical scavenging activity of L-ascorbic acid (standard sample) was 3.48 ± 0.01 *μ*g/mL, and the IC_50_ value of CLR was 44.08 ± 1.89 *μ*g/mL (Table 1).

### 3.2. Total Polyphenol and Total Flavonoid Contents

Total polyphenol content of CLR was 35.45 ± 0.06 mg/g, and total flavonoid content of CLR was 31.65 ± 0.13 mg/g (Table 2).

### 3.3. Esophageal Lesion Ratio

**Figure 2:**
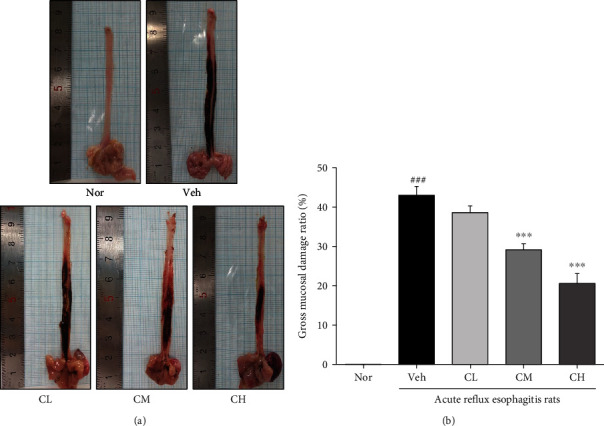
Esophagus tissue damage. The opened gross esophageal ulcer (a) and esophageal ulcer ratio (b). Nor: normal rats; Veh: water-administered to acute reflux esophagitis rats; CL: CLR 50 mg/kg body weight-administered to acute reflux esophagitis rats; CM: CLR 100 mg/kg body weight-administered to acute reflux esophagitis rats; CH: CLR 200 mg/kg body weight-administered to acute reflux esophagitis rats. Data are presented as the mean ± SD (*n* = 10). Significance: ^###^*p* < 0.001 vs. Nor group and ^∗∗∗^*p* < 0.001 vs. Veh group.

As a result of esophagus tissue damage in rats with acute reflux esophagitis, morphological changes such as various erosion and hyperemia were observed in Veh rats compared with Nor rats. And mucosal damage was found in CLR-treated rats, but was significantly decreased to CM and CH rats compared with Veh rats (Figure 2).

### 3.4. Measurement of Gastric Acid pH

**Figure 3:**
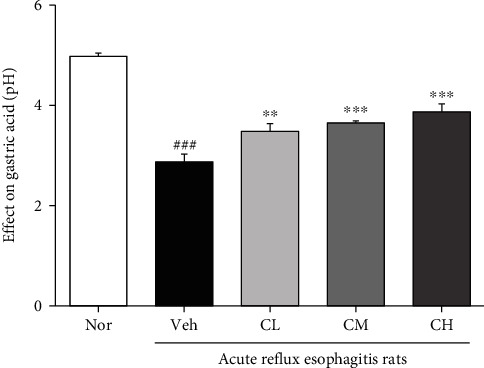
Peptic acid pH. Nor: normal rats; Veh: water-administered to acute reflux esophagitis rats; CL: CLR 50 mg/kg body weight-administered to acute reflux esophagitis rats; CM: CLR 100 mg/kg body weight-administered to acute reflux esophagitis rats; CH: CLR 200 mg/kg body weight-administered to acute reflux esophagitis rats. Data are presented as the mean ± SD (*n* = 10). Significance: ^###^*p* < 0.001 vs. Nor group and ^∗∗^*p* < 0.01, ^∗∗∗^*p* < 0.001 vs. Veh group.

The gastric acid pH of acute reflux esophagitis rats showed a significant decrease compared with Nor rats. But the gastric acid pH of CLR-treated rats was significantly increased (Figure 3).

### 3.5. Measurement of AST and ALT Levels in Serum

**Figure 4:**
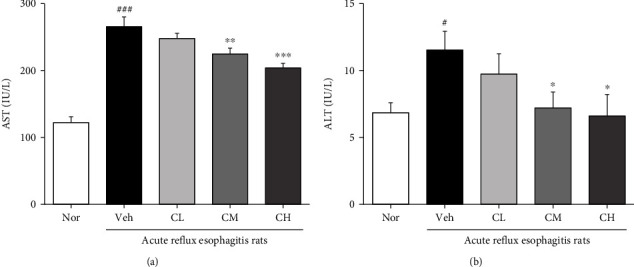
Measurement of AST and ALT levels in serum. Aspartate aminotransferase (AST) (a) and alanine aminotransferase (ALT) (b). Nor: normal rats; Veh: water-administered to acute reflux esophagitis rats; CL: CLR 50 mg/kg body weight-administered to acute reflux esophagitis rats; CM: CLR 100 mg/kg body weight-administered to acute reflux esophagitis rats; CH: CLR 200 mg/kg body weight-administered to acute reflux esophagitis rats. Data are presented as the mean ± SD (*n* = 10). Significance: ^#^*p* < 0.05, ^###^*p* < 0.001 vs. Nor group and ^∗^*p* < 0.05, ^∗∗^*p* < 0.01, ^∗∗∗^*p* < 0.001 vs. Veh group.

As a result of AST and ALT level measurement in serum, both AST and ALT of levels in serum were significantly elevated in Veh rats. And CM and CH rats were significantly reduced compared with Veh rats. Also, the levels of AST and ALT in serum were decreased in CL rats compared with Veh rats; there was no significance (Figure 4).

### 3.6. Measurement of ROS, ONOO^−^, and TBARS Levels

**Figure 5:**
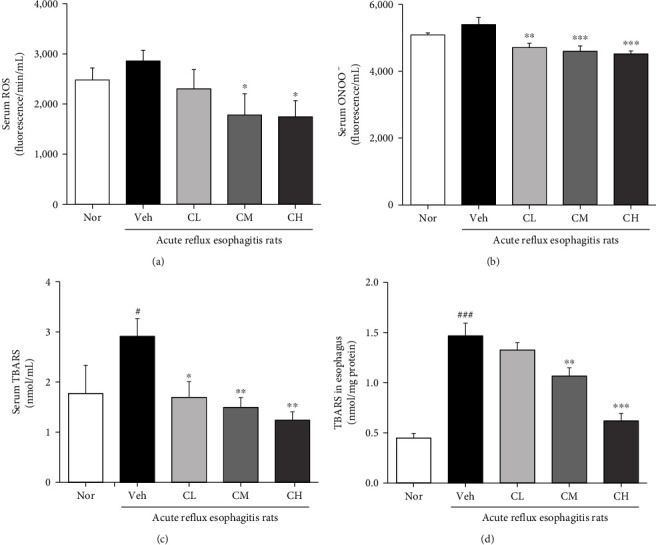
Measurement of ROS, ONOO^−^, and TBARS levels. Reactive oxygen species (ROS) (a) and peroxynitrite (ONOO^−^) (b). Nor: normal rats; Veh: water-administered to acute reflux esophagitis rats; CL: CLR 50 mg/kg body weight-administered to acute reflux esophagitis rats; CM: CLR 100 mg/kg body weight-administered to acute reflux esophagitis rats; CH: CLR 200 mg/kg body weight-administered to acute reflux esophagitis rats. Data are presented as the mean ± SD (*n* = 10). Significance: ^∗^*p* < 0.05, ^∗∗^*p* < 0.01, ^∗∗∗^*p* < 0.001 vs. Veh group.

As a result of ROS and ONOO^−^ levels measured in serum, it increased in Veh rats compared with Nor rats but not significantly. On the other hand, ROS level was significantly decreased to CM and CH rats compared with Veh rats, and also decreased in CL rats. ONOO^−^ level was also significantly decreased to all CLR-treated rats (Figures 5(a) and 5(b)).

As a result of TBARS level measurement in serum and esophagus tissue, TBARS levels of serum and esophagus tissue were significantly increased in Veh rats compared with Nor rats. In contrast, TBARS level in serum was decreased to all CLR-treated rats compared with Veh rats. Also, TBARS level in esophagus tissue was significantly decreased to CM and CH rats compared with Veh rats, and the CL rats showed to decrease (Figures 5(c) and 5(d)).

### 3.7. Expression of NADPH Oxidase Proteins

**Figure 6:**
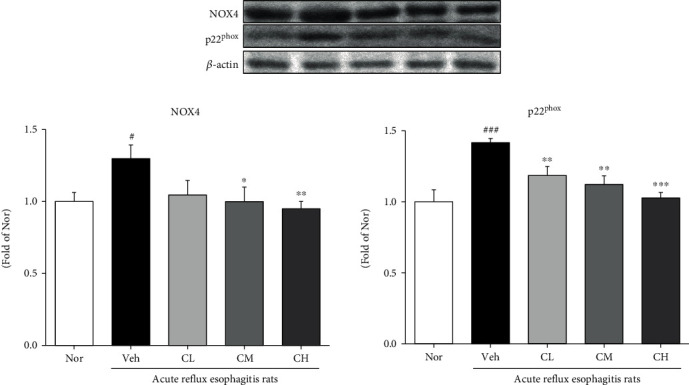
Expressions of NADPH oxidase proteins. Nor: normal rats; Veh: water-administered to acute reflux esophagitis rats; CL: CLR 50 mg/kg body weight-administered to acute reflux esophagitis rats; CM: CLR 100 mg/kg body weight-administered to acute reflux esophagitis rats; CH: CLR 200 mg/kg body weight-administered to acute reflux esophagitis rats. Data are presented as the mean ± SD (*n* = 10). Significance: ^#^*p* < 0.05, ^###^*p* < 0.001 vs. Nor group and ^∗^*p* < 0.05, ^∗∗^*p* < 0.01, ^∗∗∗^*p* < 0.001 vs. Veh group.

The change of NADPH oxidase proteins such as NOX4 and p22^phox^ was examined. Veh rats showed significantly increased expressions of NOX4 in the esophagus compared with Nor rats. However, CM and CH rats which were administrated with CLR were significantly decreased compared with Veh rats. CL rats were also downregulated compared with Veh rats. And, the p22^phox^ protein expression was significantly increased in Veh rats compared to Nor rats, whereas CLR treatment significantly downregulated p22^phox^ expression (Figure 6).

### 3.8. Expression of Antioxidation-Related Proteins

**Figure 7:**
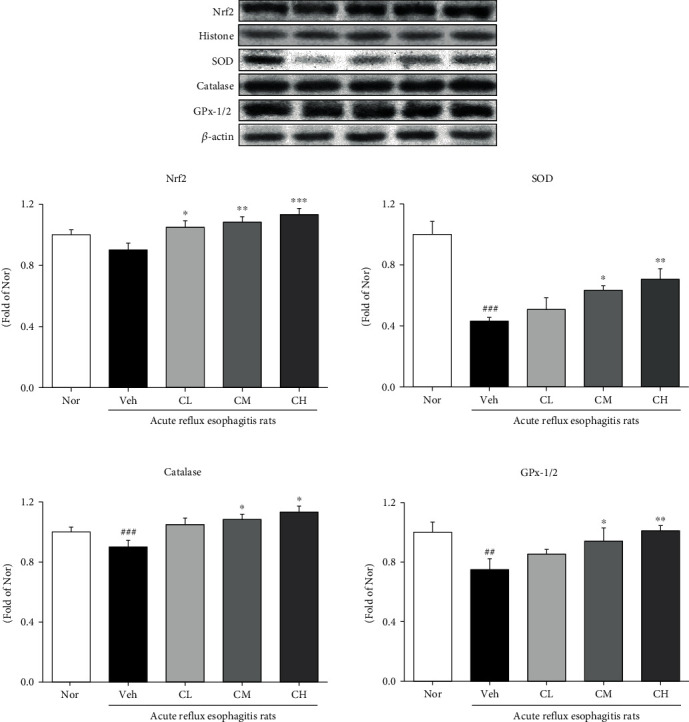
Expressions of antioxidation-related proteins. Nor: normal rats; Veh: water-administered to acute reflux esophagitis rats; CL: CLR 50 mg/kg body weight-administered to acute reflux esophagitis rats; CM: CLR 100 mg/kg body weight-administered to acute reflux esophagitis rats; CH: CLR 200 mg/kg body weight-administered to acute reflux esophagitis rats. Data are presented as the mean ± SD (*n* = 10). Significance: ^##^*p* < 0.01, ^###^*p* < 0.001 vs. Nor group and ^∗^*p* < 0.05, ^∗∗^*p* < 0.01, ^∗∗∗^*p* < 0.001 vs. Veh group.

The changes of antioxidation-related proteins such as Nrf2, SOD, catalase, and GPx-1/2 were examined. The Nrf2 protein expression was downregulated in Veh rats compared with Nor rats, whereas CLR treatment significantly increased Nrf2 expression. Moreover, Veh rats showed decreased expressions of SOD, catalase, and GPx-1/2 in the esophagus compared with Nor rats, but CM and CH rats, which were administrated with CLR, significantly increased antioxidant enzymes including SOD, catalase, and GPx-1/2 compared with Veh rats (Figure 7).

### 3.9. Expression of Mitogen-Activated Protein Kinases (MAPK)

**Figure 8:**
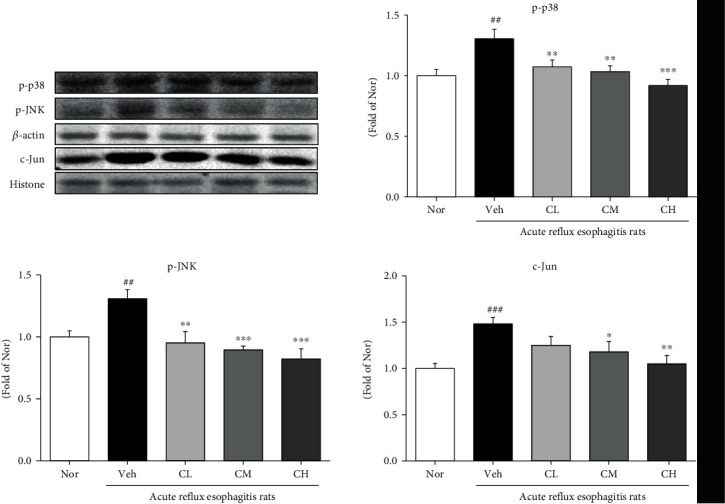
Expressions of mitogen-activated protein kinase proteins. Nor: normal rats; Veh: water-administered to acute reflux esophagitis rats; CL: CLR 50 mg/kg body weight-administered to acute reflux esophagitis rats; CM: CLR 100 mg/kg body weight-administered to acute reflux esophagitis rats; CH: CLR 200 mg/kg body weight-administered to acute reflux esophagitis rats. Data are presented as the mean ± SD (*n* = 10). Significance: ^##^*p* < 0.01, ^###^*p* < 0.001 vs. Nor group and ^∗^*p* < 0.05, ^∗∗^*p* < 0.01, ^∗∗∗^*p* < 0.001 vs. Veh group.

As a result of confirming the expression of MAPK such as p-p38, p-JNK, and c-Jun, the expressions of p-p38 and p-JNK in Veh rats were significantly upregulated compared with Nor rats. However, the administration of CLR was significantly decreased compared with Veh rats. Also, Veh rats showed increased expressions of c-Jun in the esophagus compared with Nor rats, but CM and CH rats were significantly decreased compared with Veh rats (Figure 8).

### 3.10. Expression of Proinflammatory Proteins

**Figure 9:**
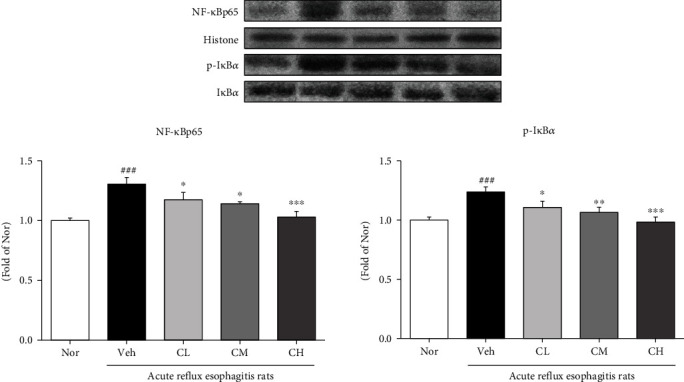
Expressions of inflammation-related proteins. Nor: normal rats; Veh: water-administered to acute reflux esophagitis rats; CL: CLR 50 mg/kg body weight-administered to acute reflux esophagitis rats; CM: CLR 100 mg/kg body weight-administered to acute reflux esophagitis rats; CH: CLR 200 mg/kg body weight-administered to acute reflux esophagitis rats. Data are presented as the mean ± SD (*n* = 10). Significance: ^###^*p* < 0.001 vs. Nor group and ^∗^*p* < 0.05, ^∗∗^*p* < 0.01, ^∗∗∗^*p* < 0.001 vs. Veh group.

**Figure 10:**
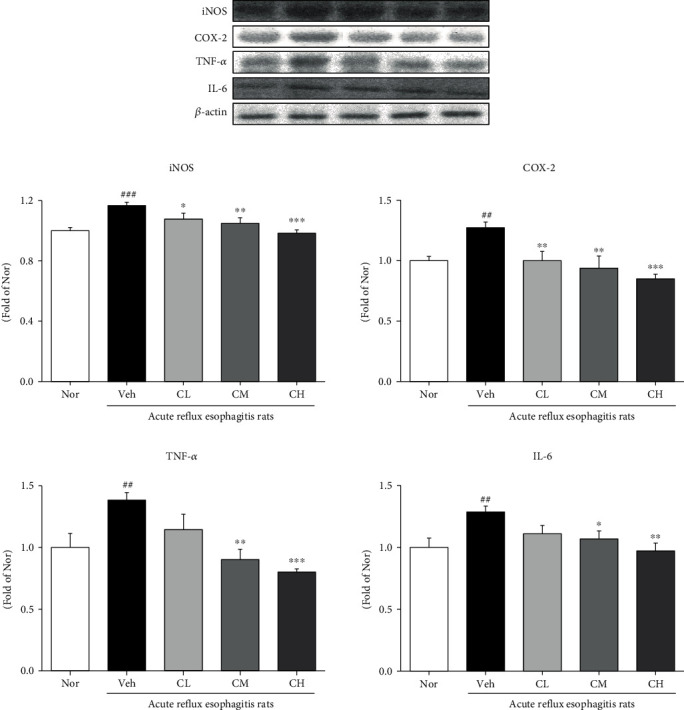
Expressions of proinflammatory enzymes and inflammatory cytokines. Nor: normal rats; Veh: water-administered to acute reflux esophagitis rats; CL: CLR 50 mg/kg body weight-administered to acute reflux esophagitis rats; CM: CLR 100 mg/kg body weight-administered to acute reflux esophagitis rats; CH: CLR 200 mg/kg body weight-administered to acute reflux esophagitis rats. Data are presented as the mean ± SD (*n* = 10). Significance: ^##^*p* < 0.01, ^###^*p* < 0.001 vs. Nor group and ^∗^*p* < 0.05, ^∗∗^*p* < 0.01, ^∗∗∗^*p* < 0.001 vs. Veh group.

The changes of inflammation-related proteins such as NF-*κ*Bp65 and p-I*κ*B*α* were examined. The expressions of NF-*κ*Bp65 and p-I*κ*B*α* in Veh rats were significantly upregulated compared with Nor rats. However, the administration of CLR was significantly decreased compared with Veh rats (Figure 9). And the changes of iNOS, COX-2, TNF-*α*, and IL-6 were examined. The expressions of iNOS and COX-2 in Veh rats were significantly upregulated compared with Nor rats. However, the administration of CLR suppressed the expressions of proinflammatory enzymes compared with Veh rats. Also, in Veh rats, the expressions of TNF-*α* and IL-6 were significantly upregulated compared with Nor rats. On the other hand, in CM and CH rats, the expressions of TNF-*α* and IL-6 were significantly downregulated by comparing to Veh rats (Figure 10).

### 3.11. Expression of Tight Junction Proteins

**Figure 11:**
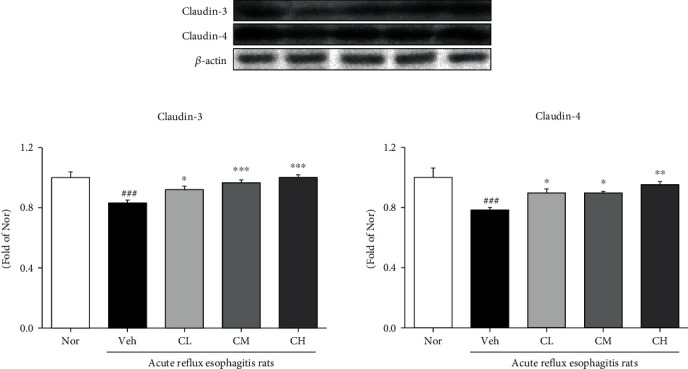
Expression of tight junction proteins. Nor: normal rats; Veh: water-administered to acute reflux esophagitis rats; CL: CLR 50 mg/kg body weight-administered to acute reflux esophagitis rats; CM: CLR 100 mg/kg body weight-administered to acute reflux esophagitis rats; CH: CLR 200 mg/kg body weight-administered to acute reflux esophagitis rats. Data are presented as the mean ± SD (*n* = 10). Significance: ^###^*p* < 0.001 vs. Nor group and ^∗^*p* < 0.05, ^∗∗^*p* < 0.01, ^∗∗∗^*p* < 0.001 vs. Veh group.

As a result of confirming the expression of tight junction proteins such as claudin-3 and claudin-4, Veh rats were significantly downregulated compared with Nor rats. In contrast, the administration of CLR was significantly upregulated compared with Veh rats (Figure 11).

### 3.12. Esophagus Histological Examination

**Figure 12:**
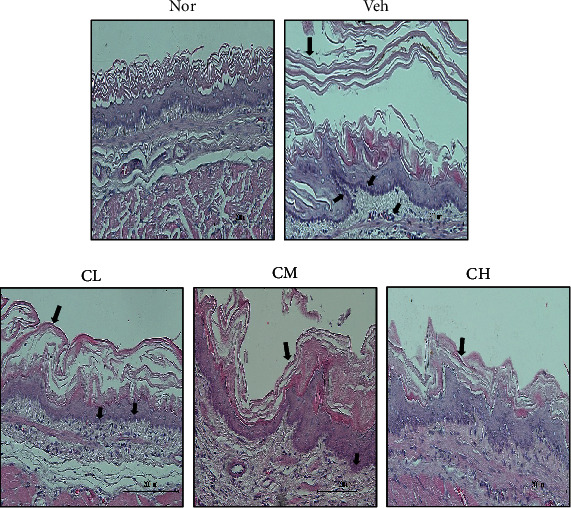
Esophagus histological examination through H&E staining. Magnification×200. Nor: normal rats; Veh: water-administered to acute reflux esophagitis rats; CL: CLR 50 mg/kg body weight-administered to acute reflux esophagitis rats; CM: CLR 100 mg/kg body weight-administered to acute reflux esophagitis rats; CH: CLR 200 mg/kg body weight-administered to acute reflux esophagitis rats.

As a result of checking esophageal status through H&E staining in the tissue of Nor rats, there were few inflammatory cells in the submucosa and squamous cell layer was normal, whereas Veh rats showed mucosal thickening and basal cell proliferation. However, CLR-treated rats had less damage to the submucosa and inflammatory cells than the Veh rats (Figure 12).

## 4. Discussion

Gastroesophageal reflux disease (GERD) is a common gastrointestinal disorder caused by abnormal reflux of gastric contents as a reflux symptom [30]. Reflux esophagitis (RE), one of the GERDs, is a common disease that causes inflammation of the esophagus due to reflux of stomach contents, causing symptoms such as heartburn, chronic cough, pharyngeal pain, and asthma [18, 31]. Proton pump inhibitors and gastric motility drugs etc. are currently used for the treatment of RE. However, these drugs have been reported to have no significant results in 20~30% of patients, and the main pathogenic factors cannot be reduced, making complete treatment difficult. In addition, it is known that serious complications occur when used for a long time [32, 33]. Therefore, in the current study, research on traditional Korea medicine that shows benefits in reducing recurrence and side effects and alleviating symptoms has been actively conducted [34–36]. So, we conducted an experiment to confirm the effect on experimentally induced acute reflux esophagitis (ARE) using Curcumae longae Rhizoma possessing the strong inhibitory effect against inflammation.

First, DPPH and ABTS free radical scavenging activities of CLR were confirmed. In addition, total polyphenol and total flavonoid contents were measured. As a result, it was shown that CLR exhibits excellent antioxidant effects (Tables 1 and 2). Based on the results, this study confirmed the protective effect of CLR against the esophageal mucosa in acute reflux esophagitis rats. The rats have increased esophageal ulcer and damage ratio compared to Nor rats, while CM and CH rats significantly reduced compared to Veh rats (Figure 2). In addition, in CLR-treated rats, the pH of gastric acid was significantly increased compared to Veh rats (Figure 3).

Reactive oxygen species (ROS) is a collective common term that includes highly oxidative radicals such as hydrogen peroxide (H_2_O_2_), hydroperoxyl radical (HO·_2_), hydroxyl (OH^−^), and superoxide (O_2_·^−^) radicals. Among them, O_2_·^−^ and H_2_O_2_ activate multiple signaling pathways that cause cell proliferation and apoptosis, elevated vascular tone, fibrosis, and inflammation, and excessive ROS cause mitochondrial damage or dysfunction that causes mitochondrial cell oxidative stress [37, 38]. Also, malondialdehyde (MDA), one of the oxidative stress (OS) markers, arises from lipid peroxidation of polyunsaturated fatty acids and can estimate the extent of lipid peroxidation [39]. Another pathway to OS is the NADPH oxidase (NOX) family, and increasing NOX is a mechanism for overproduction of OS [40]. The family of NADPH oxidases, known as NOX enzymes, is the group of transmembrane enzymes that catalyze the production of ROS from NADPH and oxygen. NOX4, one of NADPH oxidases, is found to mediate polyphenol-induced cellular senescence in endothelial cells. NOX4 inhibition and elimination are effective not only to reduce cell aging but also to reduce ROS levels [41]. In this experiment, we confirmed the inhibitory effect of CLR on oxidative stress in ARE model. It is known that in ARE, ROS is produced in mucosal epithelial cells stimulated by reflux of gastric contents, which increases esophageal mucosal damage [42]. As a result of confirming the levels of ROS and ONOO^−^, biomarkers of oxidative stress in serum, it was significantly decreased in CM and CH, and TBARS levels, lipid peroxides in serum and tissue, were also significantly decreased in CM and CH (Figures 4 and 5). In addition, administration of CLR significantly reduced NOX4 and significantly reduced p22^phox^, a regulatory isoform for NADPH oxidase (Figure 6).

High cellular levels of ROS activate the dissociation of Nrf2 from Keap-1 and its subsequent transfer to the nucleus. Nrf2 translocated into the nucleus interacts with antioxidant response elements (ARE) to modulate intracellular antioxidant responses. Thus, the Nrf2 pathway is considered a major factor regulatory mechanism for reducing oxidative stress and major regulator of the antioxidant defense system [43]. HO-1, regulated by Nrf2, is induced by a wide range of stresses such as oxidative stress and inflammatory mythology, and has a potential role in cellular antioxidant defense. And antioxidant enzymes such as SOD, catalase, and GPx protect cells from oxidative stress [44]. In this experiment, administration of CLR increased the expression of Nrf2 and HO-1, thereby increasing the expression of antioxidant-related enzymes such as SOD, catalase, and GPx-1/2 (Figure 7).

In general, ROS are known to activate MAPKs and NF-*κ*B in response to inflammatory agonists. Activated MAPKs are involved in directing cellular responses to various stimuli and in the regulation of cellular processes, such as proliferation and differentiation, cell survival, and apoptosis, and NF-*κ*B is found in many types of cells and is involved in cellular responses to various stimuli, such as stress, cytokines, oxidized low-density lipoproteins, and bacterial or viral antigens. Also, it regulates inflammation by promoting the expression of TNF-*α*, IL-6, and other proinflammatory cytokines such as COX-2 and iNOS [45–47]. Our findings reduced the expression of MAPK-related factors (such as p-p38, p-JNK, and c-Jun), and NF-*κ*B pathways NF-*κ*Bp65, p-I*κ*B*α*, proinflammatory (such as COX-2 and iNOS), and inflammatory cytokines are reduced (Figures 8 and 9). These results indicate that CLR reduces inflammation through the MAPKs and NF-*κ*B signaling pathways.

Tight junctions (TJs) are responsible for the formation of functional epithelial and endothelial barriers that regulate the passage of cells and solutes through the intercellular space as the most important structural component for the formation of constitutive barrier function in endothelial and epithelial cells. TJs include the claudin family, occludin, junctional adhesion molecules, and tricellulin, and decreased expression of its contribution to the increased permeability of the gastrointestinal epithelium in GERD [48–50]. We observed the effects of CLR of tight junction proteins of the esophageal mucosa in an ARE rat model. Our findings increased the expression of TJs (such as claudin-3 and claudin-4) in the CLR model (Figure 10).

In our findings, CLR increased the expression of antioxidant-related factors by suppressing factors related to oxidative stress. In addition, it was suggested that the reduction of oxidative stress-related factors suppresses the expression of MAPKs and NF-*κ*B pathway-related factors, thereby suppressing inflammation of the esophageal mucosa.

## 5. Conclusion

The aim of our study was to demonstrate the ameliorating effects of bioactive CLR of esophageal damage caused by ARE. Taken together our research results, CLR suppressed excessive oxidative stress generation, thereby activating the expression of the antioxidant factor by the Nrf2 pathway and inhibiting the expression of proinflammatory proteins through the MAPKs and NF-*κ*B pathways. Therefore, the results of this study suggest that CLR is a new material for the treatment of acute reflux esophagitis.

## Figures and Tables

**Table 1:** Scavenging activity of CLR on DPPH and ABTS free radical.

Sample	DPPH (IC_50_ = *μ*g/mL)	ABTS (IC_50_ = *μ*g/mL)
L-ascorbic acid	1.22 ± 0.04	3.48 ± 0.01
CLR	36.44 ± 0.76	44.08 ± 1.89

All values are expressed as the mean ± SEM of three replications.

**Table 2:** Total polyphenol and total flavonoid contents of CLR.

Sample	Total polyphenol (mg/g)	Total flavonoid (mg/g)
Curcumae longae Rhizoma 30% EtOH extract	35.45 ± 0.06	31.65 ± 0.13

All values are expressed as the mean ± SEM of three replications.

## Data Availability

The data used to support the findings of this study are included within the article.
